# Immediate Dentin Sealing: Advancing Bonding Efficacy and Clinical Success

**DOI:** 10.7759/cureus.78102

**Published:** 2025-01-27

**Authors:** Ayush Agrawal, Rohida Nehal, Krupa Gala, Sanpreet S Sachdev

**Affiliations:** 1 Conservative Dentistry and Endodontics, Bharati Vidyapeeth (Deemed to be University) Dental College and Hospital, Navi Mumbai, IND; 2 Oral Pathology and Microbiology, Bharati Vidyapeeth (Deemed to be University) Dental College and Hospital, Navi Mumbai, IND

**Keywords:** acid etching, composite restoration, conservative dentistry, dentin bonding, direct restoration, restorative dentistry

## Abstract

Immediate dentin sealing (IDS) is an innovative approach in restorative dentistry that has gained recognition for its ability to enhance bond strength, reduce sensitivity after procedures, and improve the durability of indirect restorations. This review puts together data from scientific studies to provide an in-depth analysis of IDS, focusing on its core principles, clinical advantages, applications, and challenges. Drawing from clinical trials, systematic reviews, and meta-analyses, this narrative review stresses the significance of IDS in modern adhesive protocols and its role in achieving successful and long-lasting restorative outcomes.

## Introduction and background

The quest for durable and reliable adhesive bonds in indirect restorations has led to the development of innovative protocols, among which immediate dentin sealing (IDS) stands out as a significant advancement. Traditional adhesive procedures, often involving delayed dentin sealing (DDS), expose the dentin to the oral environment and provisional materials, increasing the risk of contamination, hypersensitivity, and bond failure. IDS, first popularised by Magne (2005) [1], presents an alternative approach that involves sealing the dentin surface immediately after tooth preparation.

The IDS technique has demonstrated superior bond strength, enhanced clinical performance, and reduced hypersensitivity in indirect restorations compared to DDS [2,3]. This review provides an in-depth exploration of IDS, detailing its mechanisms, benefits, clinical applications, and future directions.

## Review

Principles of immediate dentin sealing

Hybrid Layer Formation and Stability

IDS enhances the formation and stability of the hybrid layer by infiltrating demineralized dentin with adhesive resins. This micromechanical interface acts as an anchor for indirect restorations. Unlike DDS, which delays hybridization and leaves the dentin exposed to contaminants, IDS ensures immediate infiltration and polymerization of resin adhesives, protecting the dentin structure from degradation. Deniz et al. (2021) demonstrated that IDS results in a thicker and more durable hybrid layer compared to DDS [4].

Protection From Contamination

During indirect restorative procedures, the dentin surface is often exposed to saliva, blood, and temporary cement, which compromise bond strength and hybrid layer integrity. IDS eliminates this risk by creating a protective barrier immediately after preparation [5]. Studies have shown that contamination from provisional cements significantly reduces the bonding efficacy of DDS, a challenge effectively mitigated by IDS [6,7].

Redistribution of Polymerization Shrinkage Stress

One of the critical mechanisms of IDS is the redistribution of polymerization shrinkage stress. By sealing the dentin before the cementation of the final restoration, IDS prevents stress concentrations at the adhesive interface, thereby enhancing bond durability [2]. Magne P (2005) confirmed that IDS delays the stress application on the dentin interface, resulting in improved adhesive longevity [1].

Superior Adhesive Interaction With Restorative Materials

IDS optimizes the interface between the tooth and the final restoration by creating a pre-polymerized bonding layer. This layer improves the compatibility of resin cement and restorative materials during final cementation, leading to stronger and more stable adhesive bonds. Immediate dentin sealing enables the dentin bond to mature to its full strength in an environment free from stress, eliminating the impact of polymerization stress or C-factor forces [8].

Clinical benefits of immediate dentin sealing

**Figure 1:**
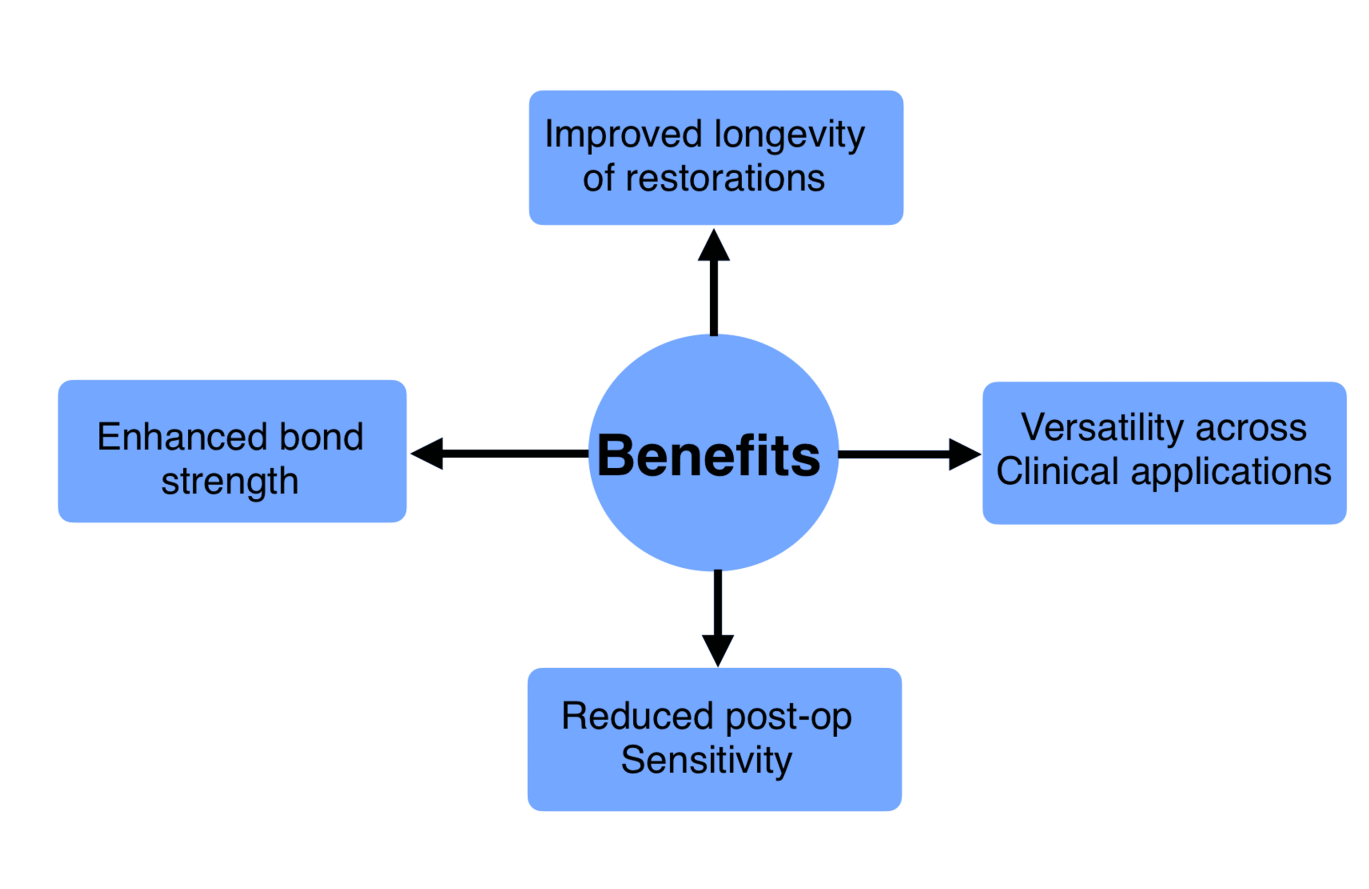
Benefits of immediate dentin sealing. Image credits: Nehal Rohida and Ayush Agrawal.

IDS has several benefits (Figure 1). Multiple studies have established the superiority of IDS in achieving higher bond strength compared to DDS. Magne et al. (2005) reported that IDS increases the resin-dentin bond strength by eliminating exposure to contaminants and allowing adhesive systems to cure in optimal conditions [2]. Falkensammer et al. (2014) further corroborated this finding, emphasizing the role of IDS in maintaining adhesive strength even under thermocycling conditions [9]. Postoperative sensitivity, often caused by fluid movement in open dentin tubules, is significantly reduced with IDS. By immediately sealing the tubules, IDS minimizes fluid dynamics and prevents hypersensitivity. Hu and Zhu (2010) observed that patients treated with IDS reported substantially lower sensitivity following cementation compared to those treated with DDS [10].

The enhanced bond strength and reduced sensitivity associated with IDS directly contribute to the longevity of indirect restorations. Gresnigt et al. (2019) conducted an 11-year clinical trial, demonstrating that ceramic veneers bonded with IDS exhibited superior performance, with minimal adhesive failures over time [11]. In areas with limited and superficial dentin exposure, the space available for the bonding agent and the restorative material is minimal. The application and curing of the bonding agent in such cases can significantly reduce the space available for the restoration. Since this can negatively impact stress distribution, immediate dentin sealing is not advised for superficial dentin exposures. However, for deeper preparations, such as those seen in Class IV or V defects or inlay, onlay, or overlay restorations, sufficient space is available for the restorative material. In such cases, immediate dentin sealing is appropriate and helps maintain a balanced thickness ratio between the restoration and the luting agent [1]. IDS prevents the formation of gaps at the tooth-restoration interface, ensuring better marginal integrity. Studies have highlighted its role in minimizing microleakage and preventing secondary caries, a common cause of restoration failure [12,13].

Clinical advantages of IDS

Numerous studies affirm that IDS enhances bond strength in indirect restorations. Research by Magne P and Deniz et al. highlights its ability to strengthen the adhesive interface, resulting in superior clinical outcomes [1,4]. Published studies further validate the long-term stability of IDS-treated bonds, especially in high-stress restorations. By sealing the dentin immediately after preparation, IDS effectively reduces postoperative sensitivity caused by exposed dentinal tubules during the provisional phase. A systematic review by Josic et al. confirmed that IDS significantly reduces hypersensitivity compared to direct dentin sealing (DDS) techniques [9].

By reducing the microleakage and protecting the adhesive layer from degradation, IDS improves the longevity of indirect restorations. Studies by Portella et al. and Magne et al. have demonstrated that IDS prolongs the clinical success of restorations, particularly in endodontically treated teeth, which are prone to adhesive failures [2,13]. Immediate dentin sealing (IDS) has proven effective across various clinical applications, including endodontically treated teeth, where it enhances adhesion to sclerotic dentin [11,14]. In computer-aided design and computer-aided manufacturing (CAD/CAM) restorations, IDS preserves bond quality even when there are delays in fabrication [7,13]. Additionally, in onlays and veneers, IDS provides a clean and sealed surface for adhesive cementation, minimizing the risk of contamination [14,15].

Clinical protocol

**Figure 2:**
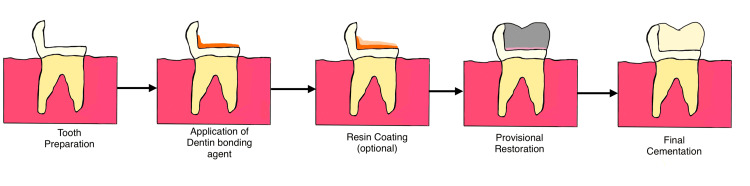
Diagrammatic representation of step-by-step procedure of immediate dentin sealing. Image credits: Nehal Rohida, Ayush Agrawal.

The clinical protocol for IDS involves various steps, as represented in Figure 2. Tooth preparation was performed using minimally invasive techniques, ensuring proper isolation to prevent contamination. For margins terminating in dentin, a well-defined chamfer of 0.7 mm to 0.8 mm was recommended to provide clear margin visibility and sufficient space for both the adhesive layer and the restoration. A shallow chamfer often resulted in adhesive resin flowing over the margin, compromising margin definition and the restoration's thickness [1].

The prepared tooth surface was cleaned with a mild etchant (e.g., 37% phosphoric acid) for 2-3 seconds to differentiate enamel from dentin. After thorough rinsing and gentle air-drying, the enamel exhibited a frosty appearance, while the dentin appeared glossy. A diamond or carbide bur was used to expose a fresh layer of dentin. Over the freshly exposed dentin, a thick layer of bonding agent was applied using a multi-layering technique and subsequently light-cured. The adhesive system was applied at its “ideal” thickness, and the manufacturer’s instructions were followed closely [2,3,14].

In cases including unfilled adhesive, an additional layer of flowable composite resin is applied over the bonded dentin to reinforce the hybrid layer and protect the adhesive during impression taking [14,15]. For impression-taking, silicone-based materials that are compatible with IDS are advised, ensuring minimal disturbance to the sealed layer. Silicone-based materials are recommended following immediate dentin sealing to ensure accurate impressions and avoid adverse interactions between the impression material and the adhesive layer [8].

For the provisional restoration, non-eugenol-based temporary cement is advised to avoid interference with the IDS layer. Before final cementation, the IDS layer was cleaned with an alcohol-based solution or gentle air abrasion to remove any surface contaminants. This was followed by applying a thin adhesive layer (without etching), luting cement, and the placement of the final restoration [2,7,13-16].

Clinical applications of IDS

IDS is widely employed in indirect restorations, including crowns, inlays, onlays, and veneers. Sealing dentin before provisionalisation ensures a more reliable and durable bond for the final restoration. Van den Breemer et al. (2019) demonstrated the clinical advantages of IDS in bonding lithium disilicate posterior crowns, reporting higher patient satisfaction and reduced hypersensitivity [16]. For minimally invasive restorations, such as ceramic laminate veneers, IDS reinforces the adhesive bond without compromising the integrity of the tooth structure. Gresnigt et al. (2019) noted the technique’s significance in achieving long-term veneer stability [11]. IDS has also been employed in composite resin inlays and onlays to enhance adhesion and reduce polymerization shrinkage stress. Shafiei et al. (2020) demonstrated the efficacy of IDS in improving the bond strength of composite resin restorations in premolars [12].

Factors influencing the success of IDS

The choice of adhesive system significantly impacts the success of IDS. Universal adhesives and dual-cure systems are often preferred for their compatibility and performance. Deniz et al. (2021) highlighted the importance of using adhesives that offer high bond strength and compatibility with luting cements [4]. Pre-treatment methods, such as chlorhexidine application or sandblasting, can improve resin infiltration and stabilize the hybrid layer. Kovalsky et al. (2022) demonstrated that sandblasting enhanced the thickness and stability of the adhesive layer in IDS [17]. Temporary cements may interfere with the IDS layer, reducing its efficacy. Silva et al. (2016) recommended the use of resin-based temporary cements to preserve the integrity of the IDS layer [6]. Thermal cycling and aging tests have shown that IDS maintains its bond strength under simulated oral conditions. Leesungbok et al. (2015) confirmed that IDS outperforms DDS in long-term thermocycling studies, demonstrating its durability [18].

Challenges and limitations of IDS

The success of IDS relies heavily on precise execution. Errors in adhesive application, curing, or handling can compromise the hybrid layer and reduce bond strength [19]. Not all adhesive systems and temporary materials are compatible with IDS. For instance, certain provisional cements may disrupt the pre-polymerized adhesive layer, leading to bond failure [6]. While short-term studies highlight the benefits of IDS, more long-term clinical trials are needed to establish its universal applicability and efficacy across diverse clinical scenarios [11,13].

Review of literature

A list of key findings on IDS from published articles is summarized in Table 1.

**Table 1:** Key findings of the recent studies published on the subject of immediate dentin sealing. IDS: immediate dentin sealing.

Author(s)	Year	Key findings relevant to the review
Portella et al. [13]	2024	Discussed whether IDS is mandatory or optional, concluding that it offers significant clinical advantages in bonding strength and hypersensitivity prevention.
Kovalsky et al. [17]	2022	Sandblasting impacts IDS layer thickness, which influences the effectiveness of the seal and overall restoration outcomes.
Samartzi TK et al. [14]	2021	A literature review of IDS concluded it is a reliable technique for enhancing bond strength and reducing dentin hypersensitivity in indirect restorations.
de Carvalho et al. [15]	2021	Reinforcing IDS with a flowable resin coating improves bonding for lightly filled adhesive systems, enhancing marginal adaptation.
Elbishari et al. [7]	2021	Summarized substantial in-vitro and emerging clinical evidence supporting IDS, including reduced post-cementation hypersensitivity and improved bond durability.
Deniz et al. [4]	2021	Chlorhexidine pretreatment with IDS enhances the shear bond strength of dual-cure adhesive cements.
Shafiei et al. [12]	2020	Proanthocyanidin-mediated IDS enhances the bond strength of composite resin inlays compared to delayed dentin sealing.
van den Breemer et al. [16]	2019	A randomized clinical trial showed IDS reduced tooth sensitivity and increased patient satisfaction in lithium disilicate posterior crowns.
Gresnigt et al. [11]	2019	A long-term clinical trial demonstrated excellent performance of ceramic laminate veneers with IDS over 11 years, showing improved restoration longevity and patient satisfaction.
Brigagão et al. [5]	2017	Compared interim cement application with immediate and delayed dentin sealing, showing higher bond strength in the IDS group.
Da Silva et al. [6]	2016	Investigated interactions between resin-based temporary materials and IDS, highlighting potential compatibility issues.
Leesungbok et al. [18]	2015	IDS significantly maintains bond strength across varying thermocycling periods, demonstrating its stability under thermal stress.
Falkensammer et al. [9]	2014	Different conditioning methods for immediate and delayed dentin sealing were compared, concluding that IDS provides superior bond strength.
Magne [3]	2014	IDS was highlighted as a critical step for tooth preparations in indirect restorations, emphasizing its technique and clinical advantages.
Hu and Zhu [10]	2010	IDS significantly reduces post-cementation hypersensitivity, proving beneficial for patient comfort in restorative procedures.
Swift [19]	2009	Critically appraised IDS as a procedure for indirect bonded restorations, concluding it offers consistent advantages in adhesive dentistry.
Magne and Nielsen [8]	2009	Explored the interactions between impression materials and IDS, emphasizing the proper selection of materials to ensure optimal bonding outcomes.
Magne [1]	2005	Introduced the concept of Immediate Dentin Sealing (IDS) and emphasized its benefits for indirect restorations, including improved bond strength and prevention of dentin hypersensitivity.
Magne et al. [2]	2005	IDS significantly improves the bond strength of indirect restorations by sealing the dentin surface prior to impression-taking.

Future directions

The development of adhesive systems tailored for IDS applications, such as bioactive adhesives and proanthocyanidin-enhanced bonding agents, could further enhance its performance [7,12]. Integrating IDS into digital workflows, including CAD/CAM systems, could streamline the procedure and improve precision in indirect restorations [11]. Future research should focus on multicentre trials to evaluate the long-term clinical outcomes of IDS in various populations and settings [13].

## Conclusions

Immediate dentin sealing represents a paradigm shift in adhesive dentistry, offering superior bond strength, reduced sensitivity, and long-term restoration durability. Despite the technique's sensitivity and compatibility challenges, IDS has proven to be a reliable and effective protocol for indirect restorations. With ongoing advancements in adhesive materials and techniques, IDS is poised to become a standard in restorative dentistry, ensuring optimal clinical outcomes and patient satisfaction.
